# Bronchitis

**Published:** 1877-03-20

**Authors:** 


### BRONCHITIS.

Dr. Russ, in the *Lancet & Observer* says: The medicinal treatment if acute bronchial inflammation should be commenced with wine of ipecacuanha, given with the view to unload the bronchi of the excessive secretion and to allay the irritability of the vagus nerve—but should only be given in the first stage. After the subsidence of the acute stage I have witnessed the best results from the following:

R.—Quinia sulphas,.........dr. iiij.
Acid phos. dil.,..........oz. ss.
Syr. Tolu,..............oz. ss.
M. Aquæ distil.,............oz. iiij.

Sig.—A dessert spoontul every four hours.

The dose should be increased or diminished according to age of the patient For the very object which is to be obtained is to produce sedation over the turgid and relaxed capillaries of the mucous tissue of the bronchi; at the same time inereasing the tonicity of the part.

When this disease assumes a still more grave form with its sequelæ, solidification, and ultimately softening of the lobules of the lung tissue with great ennervation of the system. The following should be given:

R.—Spts. vini gallici,........oz. vj.
Glycerin,...............oz. ij.
M. Tinct. Hyoscyami,......dr. iiij.

Sig.—Teaspoonful every four or five hours.

The dose to be regulated according to age of the patient.

These medicinal agents soothe the harrassing cough, aids digestion, assists assimilation, and *prevents undue tissue change.*

There is perhaps no other disease in which counter-irritation has a better effect than that of bronchial inflammation when applied so as to produce a continuous fullness of the cutaneous capilaries of the chest.

Baths or sponging with chloride of soda, with friction to the whole cutaneous surface judiciously regulated so as to keep up healthy action in the emunctories, and cause thereby elimination of the morbid products of the System, is highly indicated.

When the cough becomes so harrassing as to deprive the patient of rest—for it should be remembered that sleep has a great tendency to recuperate the enervated system; a hypodermic injection of sulph. morphia and sulph. atropia should be given at bed time. Laxatives only should be given to overcome constipation, and then the *Pul. Glycyrrhiza Comp.* of the *Prussian Pharmacopœia;* be given in doses of a teaspoonful in a wine glassful of cold water until an alvine discharge is procured. If diarrhœa exists tannate of bismuth is indicated. These measures with the indications, timely met, and the hygienic laws strictly observed and enforced throughout the whole progress of the disease, will generally be sufficient for the management of bronchial inflammation and its concomitant sequelæ.

